# Sarcomatoid carcinoma presenting as cancers of unknown primary: a clinicopathological portrait

**DOI:** 10.1186/s12885-019-6155-6

**Published:** 2019-10-17

**Authors:** Ryan W. Huey, Shalini Makawita, Lianchun Xiao, Aurelio Matamoros, Jeannelyn S. Estrella, Michael J. Overman, Gauri R. Varadhachary, Kanwal Raghav

**Affiliations:** 1 Gastrointestinal Medical Oncology, The University of Texas MD Anderson Cancer Center, 1515 Holcombe Blvd, Houston, TX 77030 USA; 2 Division of Cancer Medicine, The University of Texas MD Anderson Cancer Center, 1515 Holcombe Blvd, Houston, TX 77030 USA; 3 Biostatistics, The University of Texas MD Anderson Cancer Center, 1515 Holcombe Blvd, Houston, TX 77030 USA; 4 Diagnostic Radiology, The University of Texas MD Anderson Cancer Center, 1515 Holcombe Blvd, Houston, TX 77030 USA; 5 Anatomical Pathology, The University of Texas MD Anderson Cancer Center, 1515 Holcombe Blvd, Houston, TX 77030 USA

**Keywords:** CUP, Neoplasms, Unknown primary, Immunohistochemistry, Pathology, Molecular, Prognosis, Sarcomatoid carcinoma

## Abstract

**Background:**

Sarcomatoid carcinoma of unknown primary (SCUP) is a rare entity of either poorly differentiated carcinoma with sarcoma-like differentiation or a true mixed lineage neoplasm. Limited data regarding clinicopathological profile and management exists.

**Methods:**

We retrospectively reviewed the MD Anderson Cancer of Unknown Primary database and tumor registry to identify 48 SCUP patients between 2001 and 2017. Patient characteristics, pathology, molecular diagnostics, treatments, and outcomes were obtained. Kaplan-Meier method was used to estimate overall survival (OS) and compared using log rank test.

**Results:**

Median age at diagnosis was 59 years (range 27–86). Majority of patients were female (58%) and presented with ≥3 metastatic sites (52%), commonly lymph node (50%), bone (42%), lung (27%), and liver (21%). First line treatment included chemotherapy (35%), surgery (27%), and radiation (24%). Gemcitabine and docetaxel (18%) was the most common chemotherapy regimen. Median OS for entire cohort was 11 months (95% CI: 5.6 to 16.4). Poor performance status (PS), > 1 metastatic site, elevated lactate dehydrogenase (LDH), and high neutrophil-to-lymphocyte ratio (NLR) were significantly associated with worse OS on univariate analyses. On multivariate analyses, poor PS (HR 8.7; 95%CI: 3.0–25.0; *p* <  0.001) and high NLR (HR 3.4; 95%CI: 1.3–8.8; *p* = 0.011) emerged as independent prognostic factors for OS.

**Conclusions:**

SCUP is a rare presentation with an aggressive clinical course and limited survival. Diagnosis is difficult to make and requires careful review and synthesis of histology, immunohistochemistry, and molecular diagnostics. Chemotherapy resistance remains a challenge. Early mutational profiling is warranted, and clinical trial participation should be encouraged for this subset.

## Background

Cancer of unknown primary site (CUP) is a heterogeneous group of malignancies for which the primary site of origin cannot be identified [1]. Sarcomatoid carcinoma is a rare histologic subtype of CUP characterized by poorly differentiated carcinoma with a component of spindle cells and/or undifferentiated pleomorphic bizarre giant cells (sarcoma-like differentiation). The origin is unclear; whether these carcinomas arise from one common progenitor that has undergone divergent differentiation or a true mixed lineage neoplasm is unknown [2, 3].

Several terms have been used to describe this malignancy (See Additional file 1), including carcinoma with sarcomatoid or spindle cell features, carcinosarcoma, sarcomatoid carcinoma, spindle cell carcinoma, and carcinoma with pseudosarcomatous change [4]. Carcinomas with sarcomatoid features have been described in a number of epithelial tumors, including lung, renal cell, head and neck, urothelial, and pancreas, among others, based on the pathology and immunohistochemical analysis. These biphasic cancers may represent the act of epithelial-mesenchymal transition (EMT), in which an epithelial cell loses its polarization and assumes a mesenchymal cell phenotype, possibly driving metastatic spread [5, 6]. In most cases, the terms are determined by the anatomic primary site. For example, in the uterus and adnexa, they are grouped as mixed Müllerian tumors. In breast, the term metaplastic carcinoma is often used and in lung, they are referred to as sarcomatoid carcinomas. Pathologic challenges in classifying these subtypes and clinical uncertainties specifically related to the CUP diagnosis can create significant delays in a patient’s journey from diagnosis to receiving optimal therapy, hence, causing significant anxiety for the patients and their families.

There is limited data regarding the natural history and presentations for patients with CUP and sarcomatoid carcinoma. The purpose of this study was to characterize the clinical and pathologic characteristics, immunohistochemistry, therapeutic strategies, and survival data for patients with this rare subset of an orphan disease.

## Methods

We performed a retrospective systematic review of 48 patients who were evaluated and treated for SCUP at The University of Texas MD Anderson Cancer Center (MDACC) between 2001 and 2017. Patients were identified from a retrospective-prospective CUP database and tumor registry as described previously [7]. The study was performed under a protocol approved by the institutional review board at MDACC and a waiver for informed consent was obtained. Cases of CUP with a pathologic diagnosis of sarcomatoid carcinoma (reviewed by pathology at MDACC) were eligible. CUP was defined as presence of biopsy proven metastatic cancer without a detectable primary after an appropriate diagnostic approach incorporating clinical, pathologic, laboratory, and imaging data. Patients with sarcomatoid carcinoma from known organ sites (e.g. lung, uterus, kidney, etc.) were not included. Neoplasms that did not contain characteristics of both a sarcomatoid component and carcinoma component were excluded. We defined a historical control group by identifying 317 CUP patients without a sarcomatoid component who were evaluated and treated at MDACC between 2012 and 2015 from a prospectively managed CUP database. Baseline characteristics including age, gender, ECOG performance status (PS), number of metastatic sites, site of metastases, laboratory parameters, immunohistochemistry (IHC), and first-line treatment were collected.

### Statistical methods

Patient and tumor characteristics were summarized using descriptive statistics. Overall survival (OS) (as measured from the date of diagnosis to the date of death or last follow-up) was estimated using Kaplan-Meier product limit method and 95% confidence intervals (CI) and groups were compared using the log-rank test. Patients alive at last follow-up were censored. Factors predicting survival were identified using multivariate Cox proportion hazards models. Results were expressed in hazard ratios (HR) and 95% CI. To provide a comparison group, we took a cohort of patients (after excluding SCUP patients) from the same institutional CUP database and analyzed survival data.

## Results

### Patient and tumor characteristics

The baseline characteristics of the 48 patients analyzed are summarized in Table 1. The median age of diagnosis was 59 years (range: 27–86 years). 58% of patients were female. The majority of patients presented with 3 or more metastatic sites (52%). Common sites of metastatic disease were lymph node (50%), bone (42%), lung (27%), and liver (21%). Elevated lactate dehydrogenase (LDH) and high neutrophil-to-lymphocyte ratio (NLR) was seen in 25% and 28% of patients, respectively. First line treatment included surgery (27%), radiation (24%), and chemotherapy (35%). Among chemotherapy regimens, gemcitabine and docetaxel (18%), platinum plus taxane (12%), and gemcitabine plus platinum (9%) were the most common regimens used. Minority of patients received chemotherapy in the neoadjuvant setting (15%). Of the 13 patients who received chemotherapy as their initial treatment for whom response assessment was available, 23% had disease regression as their best response to therapy, while 77% of patients had progressive disease, with a median time to progression of 2 months (Additional file 2).

**Table 1:** Patient Characteristics

Variable	Patients N (%) (*N* = 48)
Age (years)	
Median	59
Gender	
Female	28 (58.3%)
Male	20 (41.7%)
Performance status (ECOG)	
0	8 (21.1%)
1	13 (34.2%)
2	10 (26.3%)
3+	7 (18.4%)
Number of metastatic sites	
1	15 (31.3%)
2	8 (16.7%)
3+	25 (52%)
Lactate dehydrogenase (IU/L)	
Normal (≤ 618)	33 (75%)
High (> 618)	11 (25%)
Neutrophil-lymphocyte ratio	
Low (≤ 5)	33 (71.7%)
High (> 5)	13 (28.3%)
Sites of metastasis	
Lung	13 (27.1%)
Liver	10 (20.8%)
Bone	20 (41.7%)
Lymph node	24 (50%)
First line treatment	
Surgery	9 (26.5%)
Radiation	8 (23.5%)
Gemcitabine + Docetaxel	6 (17.6%)
Taxane + Platinum	4 (11.8%)
Gemcitabine + Platinum	3 (8.8%)
Other chemotherapy	4 (11.8%)

*Note:* If numbers do not add up to 48, it is due to missing data. Percentages reflect cases with available data

### Immunohistochemistry

**Fig. 1:**
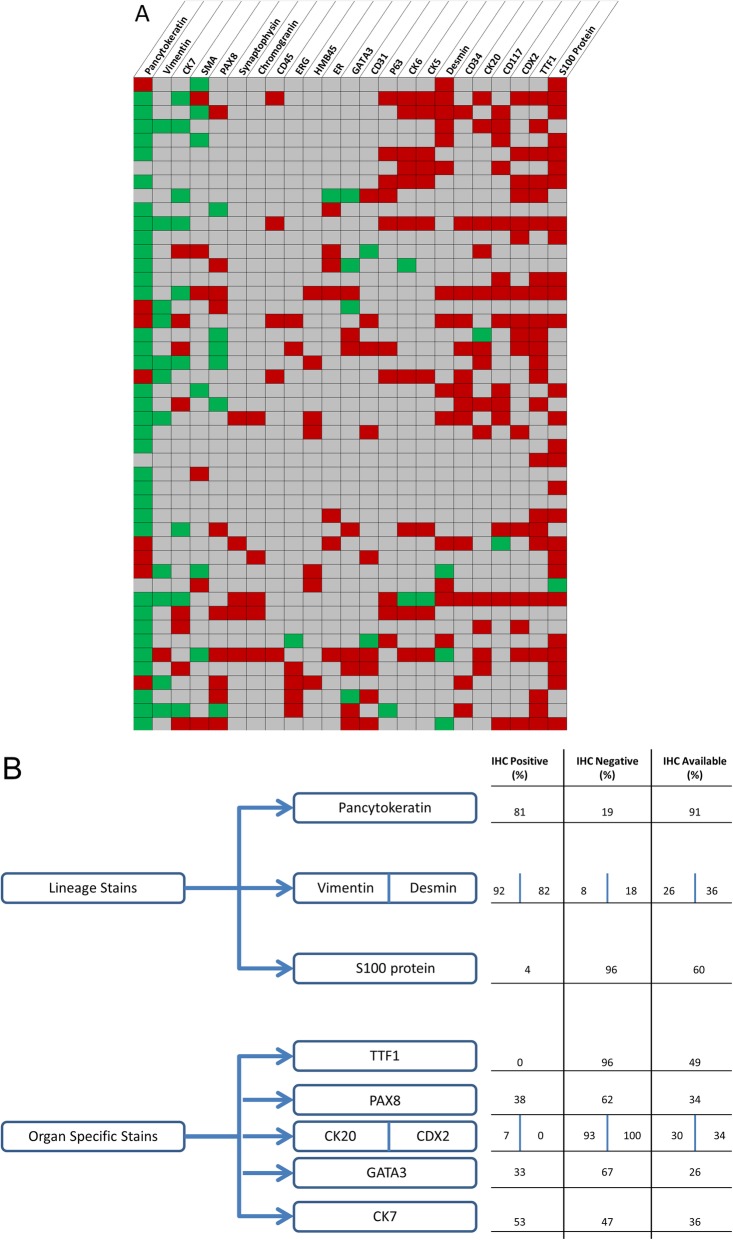
Immunohistochemistry Data. **a** Heat map of immunohistochemical (IHC) marker expression across the sarcomatoid carcinoma cases. All but one case had IHC data (*n* = 47). Twenty-three markers had IHC expression data in ≥10% of cases and are depicted. Green denotes positive staining and red denotes negative staining. Grey indicates cases where staining for that marker was not reported. **b** Lineage and organ-specific immunohistochemical markers with percentages calculated as positive and negative staining divided by the total number of cases tested. Pancytokeratin includes AE1/3 and Cam5.2 markers

To further understand the immunohistochemical characteristics of the pathology specimens, we constructed a heat map with positive and negative tests for each patient (Fig. 1a). IHC data was available for all but one case (*n* = 47). Expression was noted as positive or negative and markers expressed in ≥10% of cases were depicted (23 markers). The most common positive markers included pancytokeratin (including AE1/3 and Cam5.2; positive in 81%), Vimentin (92%), cytokeratin 7 (53%), smooth muscle actin (SMA, 50%) and PAX8 (38%). Markers noted negative in the majority of cases include S-100 protein (96%), TTF1 (100%), CDX2 (100%), CD117 (93%) cytokeratin 20 (93%) and CD34 (100%). Lineage specific marker expression is depicted in Fig. 1b with epithelial (pancytokeratin) and mesenchymal (S-100 protein, vimentin and desmin) markers as well as organ specific stains.

### Tissue of origin and mutational profiling

Tissue of Origin (ToO) testing was sent in 5 patients. ToO results showed a high probability of melanoma in 2 patients, and one patient each with sarcoma, renal cell carcinoma, and lung adenocarcinoma; however, these results were not used to change patient management in any of these cases. This likely is reflective of low patient numbers, difficult to interpret immunohistochemical data, and the severity of illness in this patient population, as some were unable to receive subsequent lines of therapy after the testing returned. The exact utility of ToO in this patient population cannot be determined from our study population.

Genomic sequencing, or next generation sequencing (NGS), was sent in 9 patients, and each patient was found to have at least one molecular alteration. However, this information was used to guide therapy in only one case, a case in which a *BRAF V600E* mutation was found and the patient was then treated with a BRAF inhibitor with the clinical suspicion of melanoma, albeit, all melanoma markers were negative on repeated testing (discussed below, case 2). In addition, several other patients had alterations that have a potential drug target, and at times, agents that were not available at the time of the patient’s treatment (See Additional file 3). Given the increase in approvals in targeted therapies, it seems likely that NGS will provide useful in this patient population with limited therapeutic options; however, more research is needed in this area.

### Univariate and multivariate survival analyses

**Fig. 2:**
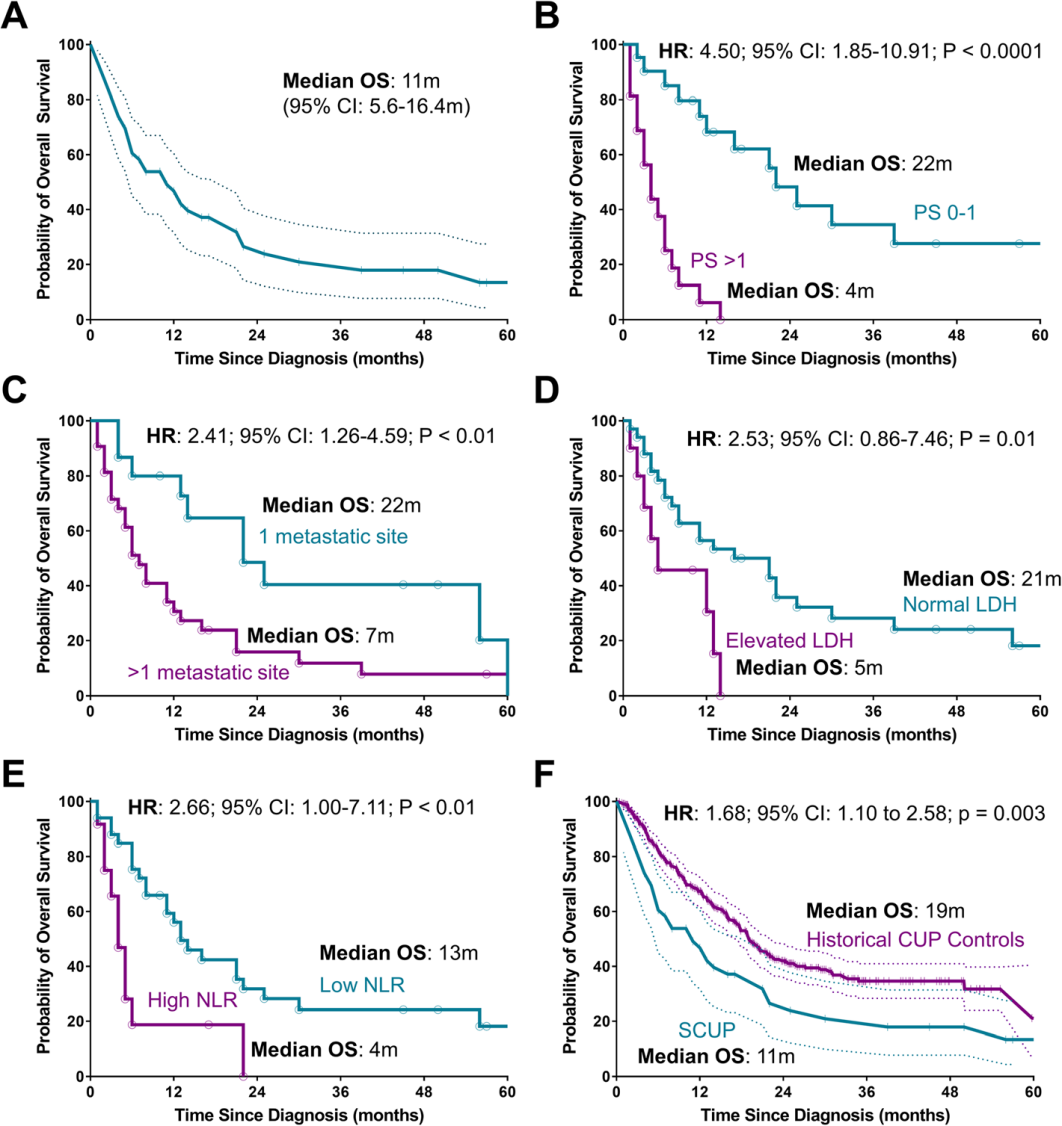
Kaplan-Meier curves of OS. Kaplan-Meier curves of overall survival **a**) All patients, **b**) By performance status, **c**) By number of metastatic sites, **d)** By LDH, **e**) By NLR, **f**) Compared to historical controls

The median follow-up for the entire cohort was 50 months. At the time of the analysis, 40 (83.3%) had died. The median OS for the entire cohort was 11 months (95% CI: 5.6 to 16.4 months) (Fig. 2). On univariate analysis, poor PS (median OS for ECOG PS ≥2: 4 months vs. PS 0–1: 22 months), number of metastatic sites (median OS for > 1 site: 7 months vs. 1 site: 22 months), elevated lactate dehydrogenase (median OS for elevated LDH 5 months vs. normal 21 months), and high NLR (high NLR 4 months vs. normal 13 months) significantly impacted OS (Fig. 2) (Table 2). On multivariate analysis, poor performance status (ECOG ≥2) (HR 8.68, 95% CI 3.01–25.02, *p* <  0.001) and elevated neutrophil-to-lymphocyte ratio (HR 3.41, 95% CI 1.32–8.77, *p* = 0.011) were associated with poor overall survival (Table 2). Patients were risk stratified using the Culine prognostic model for CUP and classified as good risk (ECOG performance status of 0 or 1 and normal LDH or no evidence of liver metastases if LDH unknown) and poor risk (ECOG performance status of 2 or more or elevated LDH or presence of liver metastases if LDH unknown) [8]. Median survival of good risk patients was 25 months compared to 4 months for poor risk patients (HR 8.87, 95% CI 3.51–22.37, *p* < 0.0001). Exploratory analysis showed the group of CUP historical controls had a median OS of 19.1 months. The hazard ratio between the SCUP group vs. the CUP controls was 1.68 (95% CI, 1.10 to 2.58, *p* = 0.003).

**Table 2:** Univariate Analyses of One-year Survival Estimates by Patient and Clinical Characteristics

Variable	Overall Survival (months)	Univariate Analysis HR / 95% CI / *P*-value			Multivariate Analysis HR / 95% CI / P-value		
ECOG Performance Status							
0–1	22	4.50	1.9–10.9	< 0.001	8.68	3.0–25.0	< 0.001
≥ 2	4						
Number of metastatic sites							
1 site	22	2.41	1.3–4.6	0.009			
> 1 sites	7						
LDH							
Normal	21	2.53	0.9–7.5	0.012			
High	5						
Neutrophil-to-lymphocyte ratio							
Low	13	2.66	1.0–7.1	0.004	3.41	1.3–8.8	0.011
High	4						

*Note:* CI, confidence interval; HR, hazard ratio; LDH, lactate dehydrogenase

### Case illustrations

The cases below illustrate the opportunities and challenges with this diagnosis and the need to work closely with pathology through the care cycle of these patients.

#### Case 1

Patient presented with abdominal fullness and early satiety. An abdominal ultrasound revealed a mass, and CT scan confirmed a large retroperitoneal mass, along with a right liver mass for which the patient underwent a distal pancreatectomy, splenectomy, right hepatectomy, subtotal gastrectomy and cholecystectomy. The resected retroperitoneal mass measured 15 × 8.9 × 8.3 cm. Pathology showed a high grade sarcomatoid malignancy involving the pancreas, liver, adrenal gland, and gastric wall. The tumor is composed of epithelioid and spindle cells with marked pleomorphism and prominent nucleoli, showed 20% tumor necrosis and exhibited a mitotic rate of 23 per 10 high power fields (HPF). Immunohistochemical stains show strong and diffuse staining for vimentin and pancytokeratin in tumor cells (Fig. 4, a&b). The tumor is negative for EMA, cytokeratin 7, cytokeratin 20, chromogranin A, synaptophysin, S-100 protein, HMB-45, desmin, SMA, EBV, CD117, DOG1, CD23, CD21, clusterin, MDM2, CD34, D240, and ERG. The resected liver mass was reported to be a poorly differentiated carcinoma. NGS showed molecular abnormalities in *BRAF*, *ERBB4*, *CDKN2A*, *PRKCI*, *TP53*, and *EPHA7*.

**Fig. 3:**
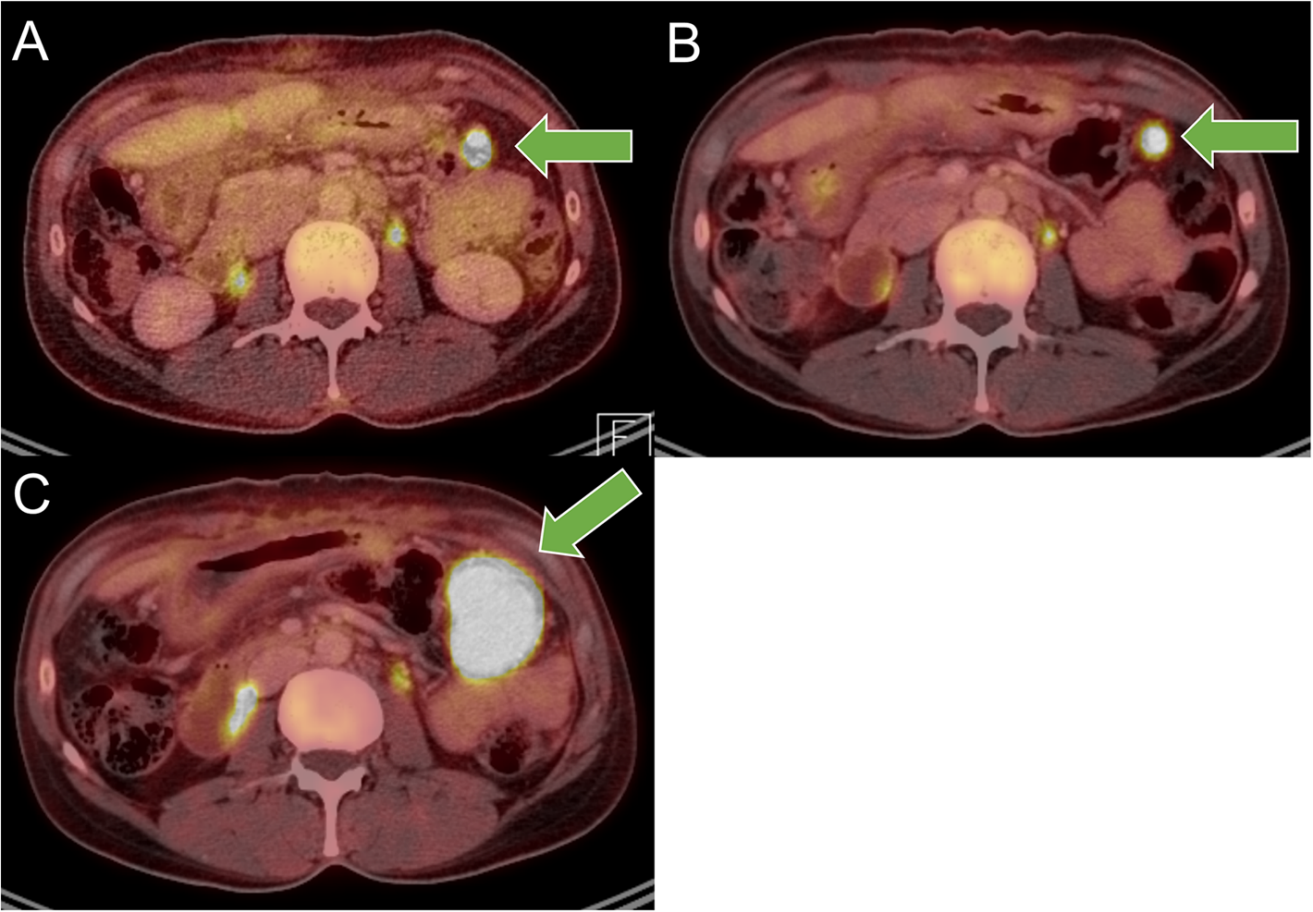
Cases illustrating sarcomatoid carcinoma of unknown primary with chemotherapy resistance. **a** PET-CT showing retroperitoneal adenopathy in the para-aortic station and indeterminate abdominal nodules. **b** PET-CT showing marginal improvement after gemcitabine and docetaxel. **c** PET-CT showing progression of disease of left upper quadrant mass

The patient subsequently presented to medical oncology for further evaluation. A PET scan of the chest, abdomen, and pelvis performed one month after surgery showed retroperitoneal adenopathy in the para-aortic station and indeterminate abdominal nodules (Fig. 3a). Given the residual (or less likely recurrent) disease, patient was started on chemotherapy with gemcitabine and docetaxel and received four cycles of this therapy with marginal improvement (Fig. 3b), but then had disease progression with enlargement of left upper quadrant mass (Fig. 3c). Patient was subsequently started on FOLFIRINOX (5-Fluorouracil, leucovorin, irinotecan, and oxaliplatin) with consideration of the pancreas or stomach being the epicenter of the primary, but experienced disease progression. Therapy was switched to a combination of doxorubicin, ifosfamide, and bevacizumab, but had continued progression and development of new liver metastases and pazopanib was considered. Patient succumbed to disease 12 months after initial diagnosis. This case demonstrates the resistance to chemotherapy that sarcomatoid carcinoma of unknown primary often demonstrates, highlighting the need to move beyond current options.

#### Case 2

Patient presented with a painless left axillary mass in the absence of other symptoms and underwent surgical resection of two lymph nodes, 2 cm and 5 cm in greatest dimension. The lymph nodes were nearly completely effaced with an high grade undifferentiated neoplasm with epithelioid and spindle cells exhibiting marked pleomorphism and prominent nucleoli growing in sheets, numerous apitypcal mitoses (mitotic rate = 23/10 high power fields) and tumor necrosis (30%). Immunohistochemical analysis was positive for vimentin and negative for all other markers including cytokeratins (OSCAR, AE1/3, 5/6 and 903), markers of muscle differentiation (desmin, caldesmon, MSA, SMA and myogenin), melanocytic markers (S-100 protein, Melan-A and SOX10), EMA, CD117, ALK-1, CD15, CD21, CD30, and CD34. A diagnosis of sarcomatoid carcinoma was made based on morphology and stains. A post-operative CT scan of the chest, abdomen and pelvis showed no evidence of disease recurrence. A PET-CT two months later showed increased uptake in the left axilla and a CT scan showed two enlarged left axillary lymph nodes (1.6 × 2.1 cm, largest in diameter) (Fig. 5a) without distant metastatic spread. Neoadjuvant gemcitabine and docetaxel was started biweekly for two months. NGS identified a *BRAF V600E* and *TP53* mutation in addition to a *KDR* germline polymorphism. In light of this molecular data, melanoma of unknown primary was entertained as the diagnosis although morphologic and immunohistochemical findings, including negative melanoma markers, did not support the diagnosis of melanoma.

**Fig. 4:**
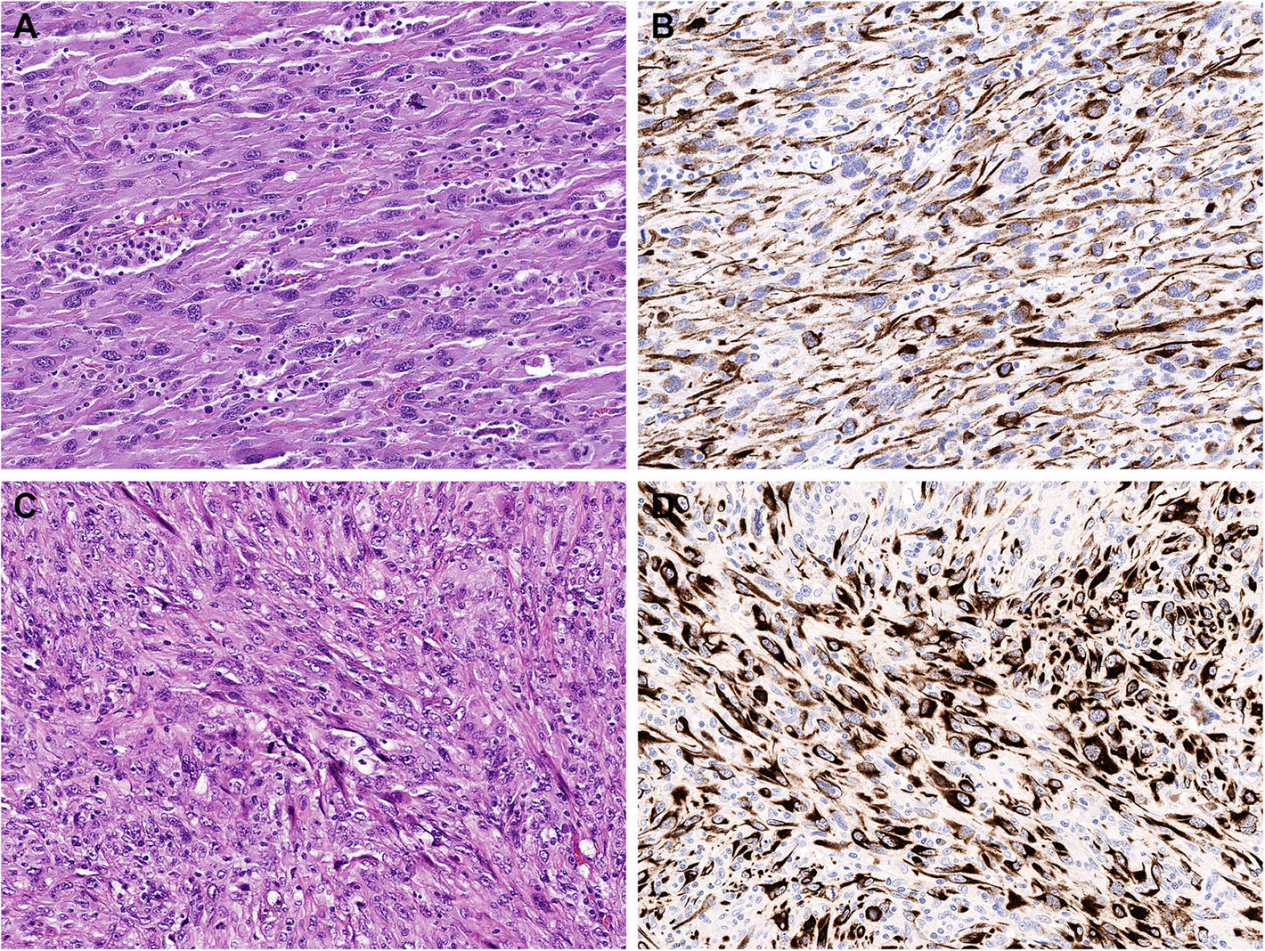
Representative pathology sections of sarcomatoid carcinoma. Representative sections of sarcomatoid carcinoma from case 1 (**a**, H&E stain and **b**, pancytokeratin stain, 20x) and case 2 (**c**, H&E stain and **d**, pancytokeratin stain, 20x). The tumors show epithelioid and spindle cells with markedly pleomorphic nuclei and large nucleoli growing in sheets and exhibiting numerous mitoses. The tumors show partial staining for pancytokeratin immunohistochemistry

**Fig. 5:**
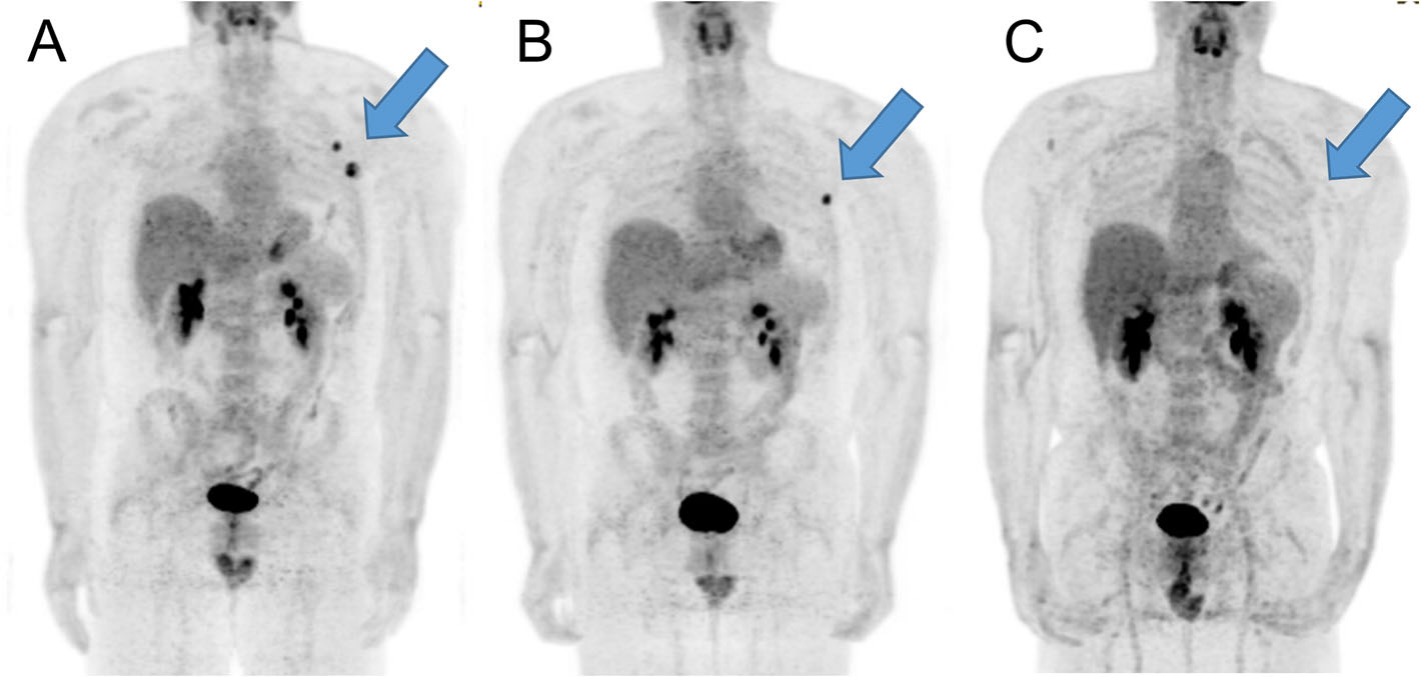
Case illustrating multimodality therapy to treat with curative intent. **a** PET-CT showing increased uptake with two left axillary nodules. **b** PET-CT showing partial response after neoadjuvant dabrafenib and four doses of ipilimumab. Note decrease in uptake in left axilla. **c** PET-CT showing no active disease at 45 months from diagnosis

Patient developed progression of disease after two months on chemotherapy and was subsequently treated with dabrafenib and four doses of ipilimumab for three months and had a partial response to therapy (Fig. 5b) followed by axillary dissection. The resected lymph nodes showed similar morphology to the previous specimen, although this tumor exhibited cytokeratin reactivity in ~ 40% of neoplastic cells (Fig. 4 c&d). As with the previous specimen, all melanocytic markers were negative. Patient completed adjuvant dabrafenib for 12 months after surgery and has been on surveillance with no evidence of disease at last follow-up, 45 months from diagnosis (Fig. 5c). This case illustrates the challenges in diagnosis and interpretation of pathology and mutational sequencing in rare CUP subtypes, and demonstrates the role of NGS, as this revealed a mutation that ultimately affected the choice of therapy. Given the limited treatment options for sarcomatoid carcinoid of unknown primary, targeted therapy may play an important role.

## Discussion

The incidence of sarcomatoid carcinoma in our CUP patient population was 4% of all CUP cases noted in our database, which is higher than previously described [2]. Although this discrepancy is likely due to referral bias in a tertiary cancer center, SCUP is a rare diagnosis within CUP. Although a small sample size, as observed with site-specific sarcomatoid carcinomas, these cancers are aggressive with median survival less than a year. Overall survival data in CUP in trials with empiric chemotherapy regimens ranges from 6 to 13 months, varying with the predominant sites of disease [9–12]. However the control group of CUP patients seen at our institution had a median OS of 19 months, suggesting that SCUP may portend a poorer prognosis. A limitation of this comparison is that the control group examined patients through 2013 and the SCUP patients were seen from 2001 to 2018.

Markers of poor prognosis noted in our SCUP report appear to be similar to other CUP subtypes or known metastatic cancers. Performance status, number/sites of metastases, and LDH have been previously described and contribute significantly to overall survival. In our study, SCUP patients with good PS (0–1), 1 site of metastasis and a normal LDH had an OS of > 20 months. Leukocyte, neutrophil and lymphocyte count and neutrophil to lymphocyte ratio (NLR) are markers of systemic inflammation, which is known to play a role in tumorigenesis [13]. A high NLR indicates an increased neutrophil count and/or a decreased lymphocyte count, as well as relative lymphopenia and NLR has prognostic value in many cancer types including prostate, lung, pancreatic cancers to name a few [14–18]. Patients with higher systemic inflammation at diagnosis may have more aggressive disease, possibly necessitating treatment more promptly, while an increasing NLR during treatment may be a precursor of disease progression and treatment failure [19]. NLR has not been previously described in CUP – in our study, patients with an elevated NLR (cutoff > 5) did significantly worse than normal NLR (4 vs. 11 months; respectively). We found it to be an important prognosticator in SCUP and it remained an independent marker on multivariate analysis.

Chemotherapy remains an important treatment modality for patients with sarcomatoid CUP, and 50% of patients received it as their first line of therapy. The most common regimen was gemcitabine and docetaxel, which has been shown to work for epithelial cancers and also sarcoma, followed by a taxane or gemcitabine combination with a platinum agent. Primary chemotherapy resistance is not uncommon and optimal regimen for sarcomatoid CUP remains to be defined. At our institution, gemcitabine and docetaxel remains the front line regimen outside a clinical trial. While debulking surgery sometimes plays a role in some sarcoma subtypes, its role in SCUP is undefined. In light of the poor prognosis of these patients, it may have a very limited role. However, consolidative surgery may be beneficial to select patients who have oligometastatic disease or good response to treatment, as shown in the case above.

Immunohistochemistry is critical to making the diagnosis of SCUP since these cases often express both broad lineage carcinoma (pancytokeratin) and sarcoma (vimentin and desmin) markers. Although IHC can be helpful in narrowing down potential tissues of origin in SCUP, the specificity and sensitivity in sarcomatoid variants (even with known primaries) may be suboptimal [20]. We therefore recommend that after using these lineage-specific stains, pathologic review entails using several tissue-specific stains to narrow the differential diagnosis further, though the heterogenous nature of sites of disease makes a specific algorithm difficult to map out.

Comprehensive genomic profiling and NGS is an emerging area in CUP [21]. A study of patients with CUP showed that comprehensive genomic profiling revealed 85% of patients with at least one clinically relevant genomic alteration that could influence therapy and a mean number of 4.2 genomic alterations per tumor [22]. The incidence of microsatellite instability-high (MSI-H) or mismatch repair deficient (dMMR) in CUP remains unknown, but this would provide another therapeutic option in an era of disease agnostic indication for pembrolizumab [23]. Trials to further evaluate the independent value of NGS on patients with CUP are ongoing. While NGS data was used infrequently to select therapy in our cohort that spans the pre-sequencing era, as new targeted therapies emerge, the role of NGS is important in this patient population with limited therapeutic options.

There has been increasing therapeutic interest in drugs that target epithelial-mesenchymal transition (EMT). As sarcomatoid carcinoma represents a biphasic cancer that may represent the act of EMT, markers specific for EMT may have important clinical implications. EMT markers are enriched in chemoresistant cell lines, which is seen in our cohort [24, 25]. It remains to be seen if EMT targeted drugs will also provide benefit to patients, though this is an area of ongoing research [26].

## Conclusions

In summary, sarcomatoid carcinoma of unknown primary is a rare presentation. Given the multiple primary sites and heterogeneous nomenclature, the diagnosis is difficult to make and requires a careful review of histology, immunohistochemistry, and molecular diagnostics. Chemotherapy resistance remains a challenge and early mutational profiling is warranted for optimal therapy planning and trial eligibility.

## Supplementary information

**Additional file 1.** Nomenclature for Sarcomatoid Carcinoma: Sarcomatoid carcinoma of unknown primary is referred to as using diverse terminology.

**Additional file 2.** Response to First Line Chemotherapy: First-line chemotherapy and reponse for 13 evaluable patients. 77% (10/13) patients had progressive disease.

**Additional file 3.** Genomic Sequencing Alterations: Proportions of alterations found using genomic sequencing of tumors of patients with SCUP, expressed as a percentage.

## Data Availability

The datasets during and/or analyzed during the current study available from the corresponding author on reasonable request.

## Abbreviations

CI: Confidence interval
CUP: Cancer of unknown primary
EMT: Epithelial-mesenchymal transition
HPF: High power field
IHC: Immunohistochemistry
LDH: Lactate dehydrogenase
MDACC: MD Anderson Cancer Center
NGS: Next generation sequencing
NLR: Neutrophil to lymphocyte ratio
OS: Overall survival
PS: Performance status
SCUP: Sarcomatoid carcinoma of unknown primary
ToO: Tissue of origin
