# Supplementary material for: Sarcomatoid carcinoma presenting as cancers of unknown primary: a clinicopathological portrait
Source: BMC Cancer. 2019 Oct 17;19:965. doi: 10.1186/s12885-019-6155-6 (PMC6796453; doi:10.1186/s12885-019-6155-6)
Supplement: Supplementary file 1 — Additional file 1. Nomenclature for Sarcomatoid Carcinoma: Sarcomatoid carcinoma of unknown primary is referred to as using diverse terminology. [file 12885_2019_6155_MOESM1_ESM.pdf]

Additional File 1

Nomenclature for Sarcomatoid Carcinoma

Sarcomatoid malignant neoplasm
Pleomorphic sarcomatoid malignant neoplasm
High grade or poorly differentiated carcinoma
Poorly differentiated carcinoma with sarcomatoid features
Unclassified spindle cell sarcomatoid neoplasm
Malignant neoplasm favor sarcoma
Sarcomatoid malignant neoplasm favor anaplastic carcinoma
Carcinosarcoma
High grade epithelioid neoplasm
Malignant spindle cell and epithelioid neoplasm
