# Supplementary material for: Sarcomatoid carcinoma presenting as cancers of unknown primary: a clinicopathological portrait
Source: BMC Cancer. 2019 Oct 17;19:965. doi: 10.1186/s12885-019-6155-6 (PMC6796453; doi:10.1186/s12885-019-6155-6)
Supplement: Supplementary file 2 — Additional file 2. Response to First Line Chemotherapy: First-line chemotherapy and reponse for 13 evaluable patients. 77% (10/13) patients had progressive disease. [file 12885_2019_6155_MOESM2_ESM.pdf]

Additional File 2

Response to First Line Chemotherapy

Patient	Chemotherapy	Best Response	Time to Progression
1	Gemcitabine + Docetaxel	PD	2 months
2	Gemcitabine + Docetaxel	PD	2 months
3	Gemcitabine + Cisplatin	PD	2 months
4	Carboplatin + Paclitaxel	PD	2 months
5	Gemcitabine + Docetaxel	PD	2 months
6	Gemcitabine + Docetaxel	PD	2 months
7	Carboplatin + Docetaxel	DR	Did not have progression. Underwent consolidative radiation.
8	Carboplatin + Etoposide	PD	2 months
9	Carboplatin + Paclitaxel	DR	Did not have progression. Underwent consolidative radiation
10	Gemcitabine + Docetaxel	PD	1 month
11	Gemcitabine + Carboplatin	DR	10 months
12	Sunitinib	PD	1.5 months
13	Doxorubicin + Ifosfamide	PD	2 months

Abbreviations: PD, Progressive disease; DR, Disease regression
