# Supplementary figures and images for: Sarcomatoid carcinoma presenting as cancers of unknown primary: a clinicopathological portrait
Source: BMC Cancer. 2019 Oct 17;19:965. doi: 10.1186/s12885-019-6155-6 (PMC6796453; doi:10.1186/s12885-019-6155-6)

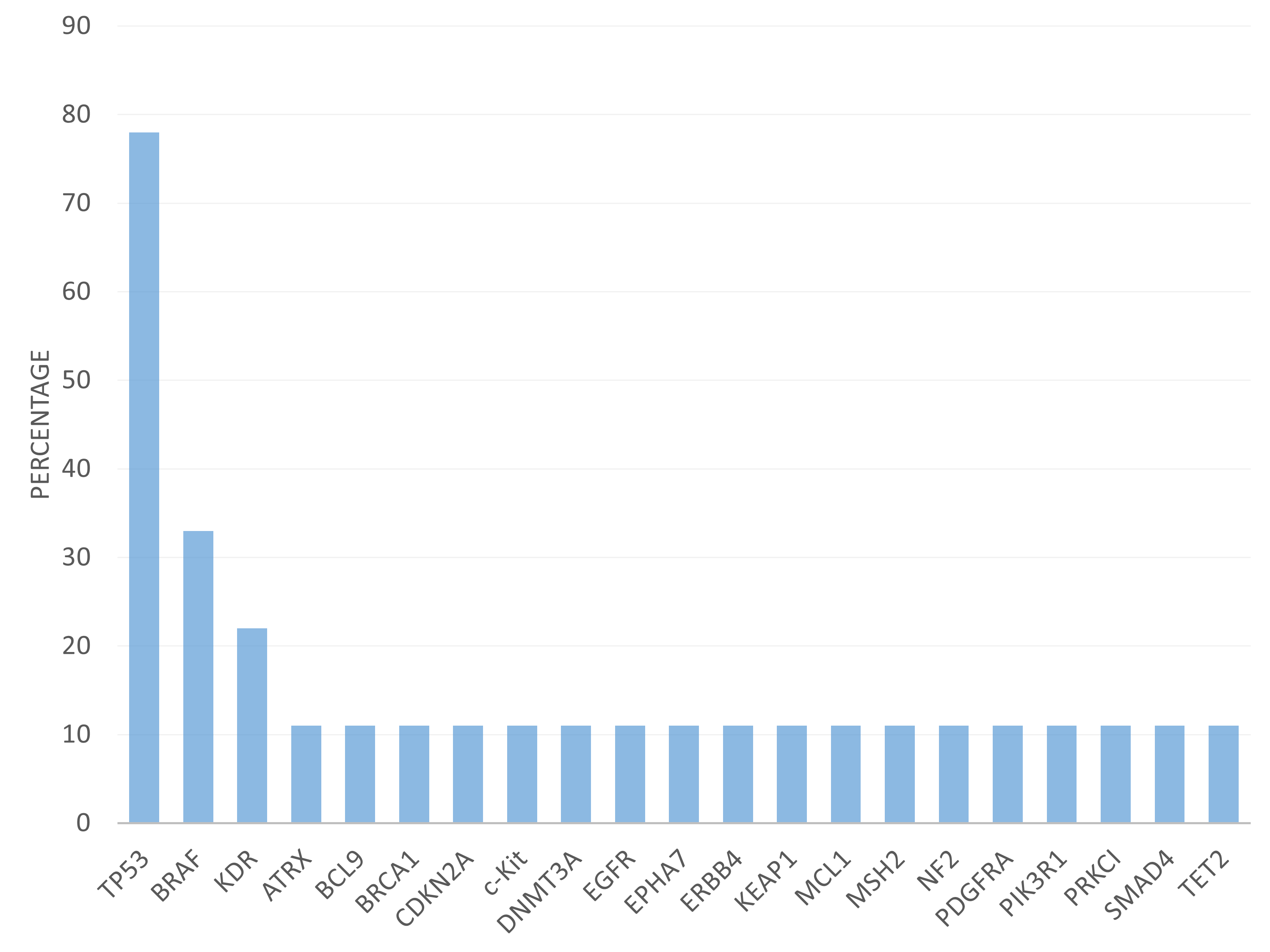

Supplement: Supplementary file 3 — Additional file 3. Genomic Sequencing Alterations: Proportions of alterations found using genomic sequencing of tumors of patients with SCUP, expressed as a percentage. [file 12885_2019_6155_MOESM3_ESM.tif]
